# Discrete Element Mesoscale Modeling of Recycled Lump Concrete under Axial Compression

**DOI:** 10.3390/ma12193140

**Published:** 2019-09-26

**Authors:** Yong Yu, Bo Wu

**Affiliations:** State Key Laboratory of Subtropical Building Science, South China University of Technology, Guangzhou 510640, China

**Keywords:** recycled lump concrete, discrete element modeling, axial compression, mechanical properties, shape of lumps, variability of mechanical performance

## Abstract

In the past decade, directly reusing large pieces of coarsely crushed concrete (referred to as demolished concrete lumps or DCLs) with fresh concrete in new construction was demonstrated as an efficient technique for the recycling of waste concrete. Previous studies investigated the mechanical properties of recycled lump concrete (RLC) containing different sizes of DCLs; however, for actual application of this kind of concrete, little information is known about the influence of the spatial locations of DCLs and coarse aggregates on the concrete strength. Moreover, the mechanical responses of such a concrete containing various shapes of DCLs are also not well illustrated. To add knowledge related to these topics, two-dimensional mesoscale simulations of RLC containing DCLs under axial compression were performed using the discrete element method. The main variables of interest were the relative strength of the new and old concrete, the distribution of the lumps and other coarse aggregates, and the shape of the lumps. In addition, the differences in compression behavior between RLC and recycled aggregate concrete were also predicted. The numerical results indicate that the influence tendency of the spatial locations of DCLs and coarse aggregate pieces on the compressive stress–strain curves for RLC is similar to that of the locations of coarse aggregates for ordinary concrete. The strength variability of RLC is generally higher than that of ordinary concrete, regardless of the relative strength of the new and old concrete included; however, variability has no monotonic trend with an increase in the lump replacement ratio. The mechanical properties of RLC in compression are little influenced by the geometric shape of DCLs as long as the ratio of the length of their long axis to short axis is smaller than 2.0. The compressive strength and elastic modulus of RLC are always superior to those of recycled aggregate concrete designed with a conventional mixing method.

## 1. Introduction

Over the past century, the human population grew exponentially from about 1500 million at the beginning of 20th century to nearly 7730 million by 2018 [1]. This great population rise promoted the construction industry to being one of the largest and most active sectors in world economy, consuming more raw materials and energy than any other economic activity [2]. Taking concrete as an example, its consumption now reaches 17.5 billion tons per year worldwide [3]. This implies that approximately 13 billion tons of sand and stone, 2.7 billion tons of cement, and 1.8 billion tons of water are consumed annually in its preparation. Another five billion tons of raw materials are also needed to produce the cement. The earth’s natural resources are limited. It is essential to seek alternative or renewable construction materials if development is to be sustained.

At the same time, about 850 million tons of construction and demolition waste (CDW) is being generated every year in the European Union (EU) alone, accounting for nearly 31% of the overall waste generation [4]. Commonly, CDW becomes landfill; however, burying it is expensive and deleterious for the environment. Significant effort is, therefore, devoted to finding effective approaches for recycling CDW. The goal is to reduce the landfill utilization and provide inexpensive construction materials [5,6]. Reusing waste concrete is considered a promising approach. Crushed waste concrete is used to partly or totally replace virgin aggregate, producing recycled aggregate concrete (RAC) [7,8]. It can efficiently reduce the consumption of natural aggregate and the problems of mining it.

Research on RAC was conducted for a long time. It started with basic observations of the effects of using recycled aggregate (RA) on the compressive strength of concrete, as well as its economic feasibility [9]. Since then, research on RAC became an area of considerable interest worldwide. Many investigations documented its durability, fatigue properties, and micro-structure [10,11,12,13,14,15,16]. The test results showed RAC’s promise and environmental benefits.

Compared with natural aggregate concrete, the performance of RAC is generally a little poorer in some respects. This is mainly due to the particular characteristics of the RA [17]. Often, a large amount of old mortar matrix adheres to the aggregate’s surface. It typically contains a large volume of micro-cracks and pores produced during the crushing procedure. Additionally, there are abundant interfacial transition zones (ITZs) between old aggregate and old mortar. These features commonly negatively affect RAC’s mechanical properties and durability; however, it was demonstrated that, after some special treatment [18,19] or simply altering the concrete mix proportions [20,21], the properties of RAC can be significantly improved.

It must be acknowledged, however, that the actual application of RAC in construction significantly lagged behind the scientific research [22]. Taking the EU as an example, currently less than 1% of RA is used in structural concrete applications [23]. The reasons hampering its wider use are multifold [24], but the most important one may be that better dismantling and demolition, crushing, and milling techniques are needed to manufacture high-quality RA cost-effectively [25]. Large-scale production of RA must be faster, easier, and cheaper.

**Figure 1:**
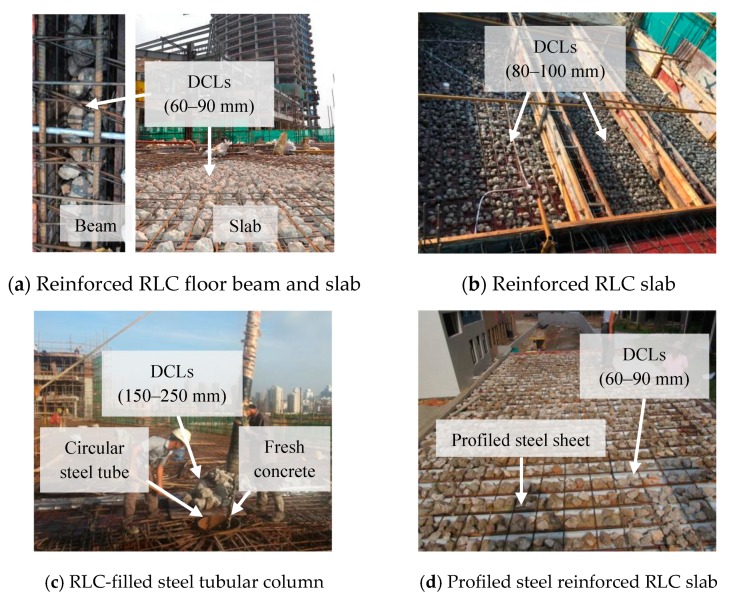
Examples of the application of recycled lump concrete (RLC) containing demolished concrete lumps (DCLs): (**a**) reinforced RLC floor beam and slab; (**b**) reinforced RLC slab; (**c**) RLC-filled steel tubular column; (**d**) profiled steel-reinforced RLC slab.

A step in this direction is a method which requires only that waste concrete be coarsely crushed into large lumps (referred to as demolished concrete lumps or DCLs) for direct mixing with fresh concrete during casting [26]. RA pieces are commonly smaller than 31.5 mm in diameter, but DCLs usually range in size from 60 to 300 mm. That makes the crushing and sieving much simpler. It allows a higher recycling ratio, because the old mortar matrix is re-used in the new concrete mixing, and less heat of hydration is released because less new cement is employed [27]. Various studies documented the performance of such recycled lump concrete (RLC, also previously referred to as compound concrete). Its compression, flexural tensile, and split tensile strength were demonstrated, as well as its creep behavior [28,29,30,31,32,33]. Tests of structural members containing DCLs investigated their static performance and disaster resistance [34,35,36]. More recently, the fatigue performance of this material in response to cyclic compression was also investigated [37]. Taken together, those tests demonstrated that the mechanical properties and structural performance of the recycled lump concrete members are similar to or only slightly inferior to those of ordinary concrete members. It is, therefore, feasible to use recycled lump concrete in construction. Some examples are presented in Figure 1.

Nevertheless, experience shows that more research is needed to better understand how best to apply this type of concrete. For instance, previous work on the uniaxial compressive behavior of recycled lump concrete mostly concentrated on the influences of the characteristic size and the replacement ratio of the DCLs, as these factors are the main difference between recycled lump concrete and the ordinary kind. To date, relatively little effort was directed toward investigating the influence of the spatial distributions of DCLs and coarse aggregates on the material’s mechanical behavior. Ordinary concrete is simply aggregate, cement paste, and ITZs connecting the two. The mesoscale structure of recycled lump concrete is more complicated. There are at least eight types of mesoscale phases when DCLs are incorporated into fresh concrete: new aggregate, new mortar, ITZs connecting the new aggregate and new mortar, old aggregate, old mortar, old aggregate–old mortar ITZs, old mortar–new mortar ITZs, and old aggregate–new mortar ITZs. Of course, the macroscopic mechanical properties of the recycled lump concrete are strongly linked with the mesoscale behavior of the constituent materials. The spatial distributions of DCLs and aggregate pieces was the primary focus of this study. This is mainly because, as the locations of DCLs and aggregates change, the spatial distributions of the eight mesoscale phases change accordingly, which may directly influence the variability of the recycled lump concrete’s mechanical properties.

Additionally, it is also significant that the geometric shapes of the DCLs are diverse but always irregular. The longest axis of a lump can vary from 1.0 to 1.7 times its characteristic size, while the shortest axis can range from 0.6 to 1.0. However, the influence of lump shape on recycled lump concrete’s mechanical properties was never documented in a published study.

This study, therefore, had two primary objectives. One was to reach a fuller understanding of the impact of the distribution of DCLs and coarse aggregate on the mechanical properties of recycled lump concrete. The other was to systematically document the effect of lump shape on recycled lump concrete’s mechanical responses. Furthermore, the differences in compression behavior between RLC and recycled aggregate concrete were also documented. However, unlike most of the previous work in this area, the mechanical behavior was investigated using a discrete element method. That technique offered the following advantages: it could easily represent the various types of interfacial transition zones, which are known to be the material’s weakest regions and are generally very important in brittle materials, and it could visually express nonlinear damage and cracking processes in the concrete [38,39,40].

## 2. A Mesoscale Multiphase Model

At the current stage, the widely employed approach to establish the concrete’s mesoscale model is the “take and place” technique [41]. The main steps involved are (a) to assume a shape for the aggregate, then calculate the amount of aggregate according to the area of the zone to be thrown, the volume percentage of aggregate in the concrete, and the aggregate’s gradation curve; (b) to generate a sequence of aggregates with specified shapes and sizes; and then (c) to place those aggregates within the thrown zone using a circle or convex polygon’s contact algorithm. To improve the production efficiency, larger aggregate commonly has priority in being placed. This procedure generally yields a desirable aggregate shape and size distribution, but it also has several disadvantages. One of the biggest problems is its efficiency. Since the preplaced aggregate usually occupies a considerable amount of space, that makes throwing the subsequent particles into the domain difficult. To solve this problem, the “take and place” technique usually should be supplemented with a special optimization algorithm [42]. This can lessen the difficulty, but naturally decrease the method’s applicability.

Consider instead an explicit kinematics-based method. Its intent is to conveniently build the mesoscale structures of ordinary concrete and recycled lump concrete. Such a method is implemented on the discrete element package Particle Flow Code 2D (PFC2D) [43]. In this discussion, all of the concrete prisms of interest can be simplified as two-dimensional 100 mm × 200 mm or 200 mm × 400 mm rectangles. The size distribution of the aggregate is assumed to follow the classic Walraven formulation [38].

(1) $$\begin{array}{c} P_{c}(D<D_{0})=P_{k}(1.065D_{0}^{0.5}D_{\max}^{-0.5}-0.053D_{0}^{4}D_{\max}^{-4}-0.012D_{0}^{6}D_{\max}^{-6}-\\ 0.0045D_{0}^{8}D_{\max}^{-8}-0.0025D_{0}^{10}D_{\max}^{-10}) \end{array}$$

where Pc(*D* < *D*_0_) denotes the area percentage of aggregate with a particle size less than *D*_0_, Pk represents the fraction of the total area occupied by aggregate pieces (usually about 75% according to the research published by Zhang and his colleagues [38]), and Dmax is the maximum aggregate size, assumed to be 25 mm [3].

To simplify the discrete element mesh and to reduce the computation load, fine aggregate less than 5 mm in effective diameter was not explicitly simulated. Instead, the fine material was merged with the cement to form the mortar phase. Table 1 presents the aggregate size distribution in a conventional concrete specimen’s mesoscopic model. For simplicity, all of the aggregate pieces were assumed to be circular in the simulations. Before placing the aggregate, the quantities of aggregate particles were calculated using the information presented in Table 1.

Taking a 200 mm × 400 mm ordinary concrete specimen as an example, the specific steps in generating its random aggregate model can be summarized as follows:

(1) Maintain the rectangle’s width as 200 mm, but enlarge its height from 400 to 800 mm to form a new 200 mm × 800 mm rectangular zone. The PFC2D software’s “wall generate” command is then used to create four boundary walls.

(2) Keeping the particle number unchanged, amplify each aggregate piece’s diameter to 1.05 times its original size.

(3) Utilize the built-in “ball generate” command to place aggregate particles in the rectangular zone. Note that the “ball generate” command automatically ignores hard-to-place particles if the aggregate content is too high [43]. In fact, in this study’s simulations, due to the artificial amplification of the thrown domain, the placement of all particles was always easy.

(4) Give each particle a very high normal stiffness, then force the top and bottom walls to move in opposite directions. This movement stops when the distance separating the two walls approaches 400 mm. With the motion of the walls, the aggregate particles also translate; however, due to their relatively high stiffness, the overlaps between adjacent particles are extremely small.

(5) In the last step, distances among aggregate pieces and distances between the pieces and the walls are adjusted. This involves giving each particle a velocity in an arbitrary direction and letting all of the particles move freely and, keeping each particle’s center unchanged, reducing its diameter to the original size shown in Table 1. This yields an optimized mesoscale structure.

The main procedure in building a mesoscale model of recycled lump concrete has two steps.

**Figure 2:**
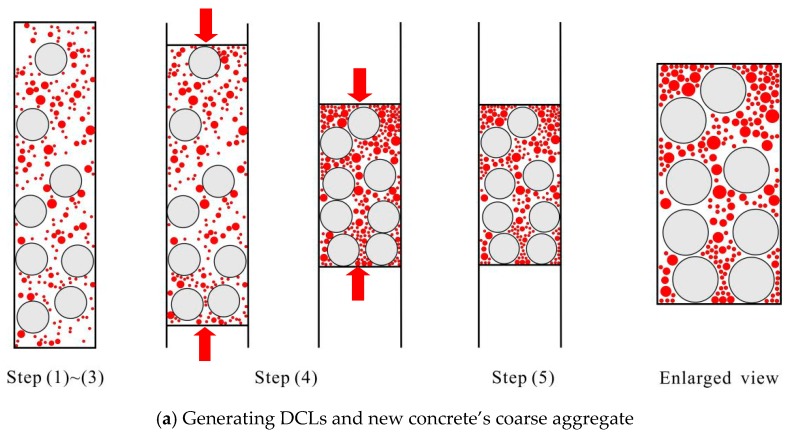

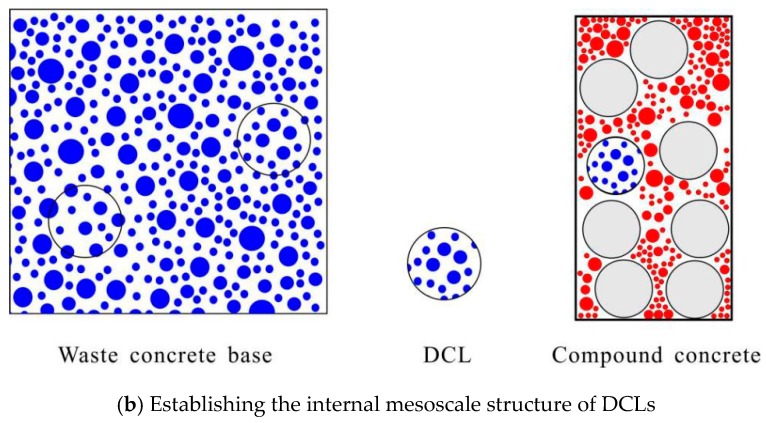
Generating a mesoscale model for recycled lump concrete.

(1) In the first step, the mesoscale structure of each DCL is temporarily neglected. The concrete lump is simply idealized as a circle or ellipse according to its characteristic size and the ratio of the long axis to short axis. The amount of coarse aggregates needed for the fresh concrete can be calculated after deducting all of the DCLs from the volume being represented. The DCLs and aggregate particles can then be thrown into the rectangles as previously described.

(2) The main purpose of the second step is to establish a mesoscale structure for the DCLs. Applying the random aggregate method for representing conventional concrete, a 1000 mm × 1000 mm concrete base can easily be generated. Circles or ellipses can then be randomly thrown into the base. After cutting any coarse aggregate intersecting with the circles and ellipses, the mesoscale structure of DCLs can be acquired. Figure 2 illustrates the internal mesoscale structure of a recycled lump concrete specimen. The replacement ratio in this example is 44%, and the average characteristic size of the DCLs is 75 mm.

## 3. The Discrete Element Method

Unlike the finite element method, the computation domain in a discrete element method (DEM) model is commonly discretized as rigid blocks or particles [39]. Through connecting them with springs, dampers, gaps, etc., the rigid elements are allowed to move dependently to simulate, for example, the nucleation, growth, and coalescence of cracks in a concrete sample under external load. The discrete element analyses conducted in this study relied on the PFC2D package. That software generally uses an explicit time-domain integration method to solve the equations of motion of rigid particles in contact [43].

**Figure 3:**
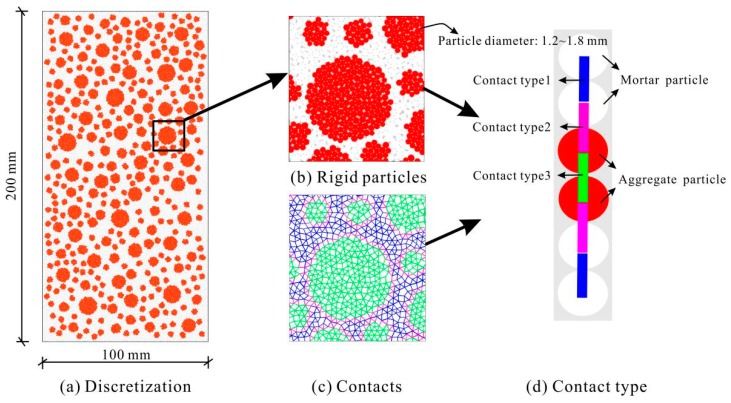
Discrete element modeling of ordinary concrete.

The concrete prisms tested in this study’s uniaxial compression tests were simplified as rectangles in modeling, and circular particles were adopted to discretize the rectangular domains. The average radius was set as 1.5 mm, and the maximum-to-minimum radius ratio was assumed to be 1.5. Additionally, since gaps are unavoidable when using circular particles to discretize a rectangle, the initial porosity of the concrete sample was set to 8.0% in advance, a value adopted in previous studies [38]. After the discretization, each particle in a discrete element model should be given appropriate properties. This was done by distinguishing the phase category of each particle using a background mesh method [44]. Figure 3 shows the rigid particles and their contacts in a 200 mm × 400 mm discrete element model of ordinary concrete. The total number of rigid particles and contacts in this model are about 4.11 × 10^4^ and 11.3 × 10^4^, respectively.

**Figure 4:**
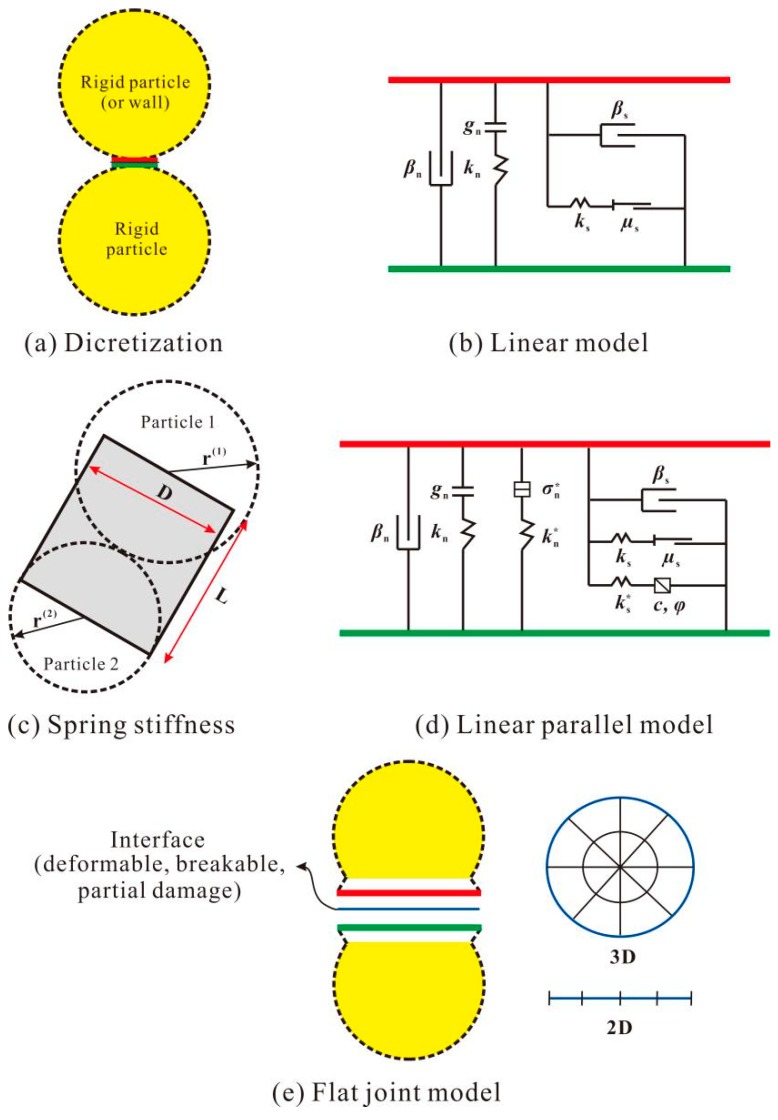
The PFC2D software’s constitutive models.

In the PFC2D software, mutual interactions among discrete particles (see Figure 4a) are usually described using contact models like those of a linear model, a linear parallel model, a flat joint model, and so on. In this study’s modeling, the linear model was adopted to simulate the interactions between a concrete sample and the loading platens of a press, and the flat joint model was used to consider the interactions among rigid particles [38].

Figure 4b gives an illustration of the linear model. As the figure shows, springs, dampers, gap cells, and friction modules are employed to simulate the relative normal and shear motions among rigid particles. The function of the gap cell is to control the activity of the normal and shear springs, limiting them to normal displacement. If its value exceeds the threshold value *g*_n_, both the normal and shear springs would enter an invalid state where they cannot resist any tensile or shear loads. The friction module is used to dissipate the friction energy. Its capacity is correlated with the friction coefficient *μ*_s_ and the compressive load.

The linear model has six main parameters: *k*_n_, *k*_s_, *g*_n_, *μ*_s_, *β*_n_ and *β*_s_ or E^*^, k^*^, *g*_n_, *μ*_s_, *β*_n_ and *β*_s_ (for details on the meanings of *β*_n_ and *β*_s_, see Table 2). Based on the truss principle presented in Figure 4c, the stiffness of the normal spring *k*_n_ can be related to the equivalent elastic modulus E^*^ as

(2) $$k_{n}=\frac{AE^{*}}{L}=\frac{(Dt)E^{*}}{L}.$$

In a plain stress state, the equivalent truss thickness *t* holds *k*_n_ to 1.0.

When the truss lies between a rigid particle and the wall, its width *D* and length *L* can be determined through

(3) $$D=2r^{\left(1\right)},\;L=r^{\left(1\right)}.$$

When the truss lies between rigid particles, the following equations hold:

(4) $$D=2\min\{r^{\left(1\right)},\;r^{\left(2\right)}\},\;L=r^{\left(1\right)}+r^{\left(2\right)}.$$

The normal–shear stiffness ratio is

(5) $$k^{*}=k_{n}/k_{s}.$$

In this study’s modeling, the normal elastic modulus E^*^ was set to 200 GPa, the elastic modulus of the loading platen [40]. Additionally, since there is normally a friction restraint between the concrete sample and the loading platens of the press, the friction coefficients between the rigid particles and the top and bottom walls were both set as 0.60, a value adopted in previous studies [45] investigating the uniaxial compressive behavior of concrete.

Figure 4d shows the main components of the linear parallel model. Two extra parallel springs are added compared with the linear model. Unlike the springs in the linear model, those parallel springs can only resist external loads of limited magnitude. Their capacities are controlled by the tensile strength *σ*_t_, the cohesive strength *c*, and the friction angle *φ.*

As explained, the interaction among adjacent particles in a two-dimensional plane is considered as a point-to-point contact in both the linear model and the linear parallel model. However, such an interaction in the flat joint model is treated as line–line. As depicted in Figure 4e, the flat joint model divides the contact line into several separate elements. Before loading, each element can independently be in a deformable, partial damage, or breakage state. As the external load increases, elements initially in a deformable state follow the linear parallel model, while elements in a breakage state follow the linear model. Table 2 presents the main parameters in the flat joint model.

Before conducting the simulations, the range for each parameter used in previous published studies [38,40,45,46,47,48,49] was compiled. Several parameters in the flat joint model were then assigned the most commonly used values (see Table 2). E^*^, *σ*_t_, and *c* were calibrated through axial compression and tensile tests using published testing protocols [49].

## 4. Discrete Element Modeling of Ordinary Concrete under Compression

### 4.1. Model Validation

To check the correctness of using discrete element modeling, the compressive tests of mortar and concrete samples reported in the literature [50] were simulated. As illustrated in that study, both the mortar and concrete samples in the tests had geometric dimensions of 100 mm × 100 mm × 200 mm, and the cement type, the sand’s fineness modulus, and the maximum size of coarse aggregates in these samples were P.O. 42.5, 2.4, and 25 mm, respectively. Table 3 lists the mix proportions and measured mechanical properties for the mortar and concrete prisms. Since the compression test reports did not provide an elastic modulus for the mortar, the value listed in the table was estimated using some published empirical formulas [51].

During the numerical analysis of the mortar sample, because that material is relatively homogeneous, only mortar–mortar contact was considered. Fully describing the mesoscale behavior of mortar–mortar contact involves the three parameters E*, *σ*_t_, and *c*. Through iterative trial calculations and adjustment, their values were determined as 21.0 GPa, 5.6 MPa, and 15.0 MPa, respectively. Using those values, the discrete element model correctly reflected the compression test data, and the calculated mechanical properties also agreed well with the test results. This can be seen in Table 3, where the errors in the predicted compressive strength and elastic modulus for the mortar were only −3.3% and −1.6%, respectively.

Concrete is normally discretized as aggregate and mortar in discrete element simulations. This implies three types of contacts: aggregate–aggregate contact, mortar–mortar contact, and aggregate–mortar contact (shown in Figure 3). The mesoscale responses of all three types are best described using flat joint models. The values of the parameters are summarized in Table 4. The aggregate–mortar contact values are *k* times those of the mortar–mortar contact because nanoindentation tests of concrete demonstrated that the aggregate–mortar ITZs are positively correlated with those of the mortar.

In the experiments reported in the literature [50], the concrete and the mortar had the same water–cement ratio; thus, the mesoscale parameters E*, *σ*_t_, and *c* for mortar–mortar contact in the discrete element model were taken as 21.0 GPa, 5.6 MPa, and 15.0 MPa, respectively. Additionally, the value of *k* (Table 4) was set to 0.80 based on test results reported by Xiao and his colleagues [13]. They reported that the ITZ varies little from 0.80 times that of the mortar regardless of the concrete’s water-to-cement ratio.

The mesoscale parameters of the aggregate–aggregate contacts were determined using extensive trial and error. With an E* of 147.0 GPa, a *σ*_t_ of 39.2 MPa, and a *c* of 105.0 MPa, the discrete element model correctly reflected the compressive behavior of the concrete samples. The calculated compressive strength and elastic modulus were then 48.30 MPa and 33.41 GPa, respectively, which were just 1.8% and 1.6% lower than the measured values. Thus, these values were used in the subsequent numerical analyses.

**Figure 5:**
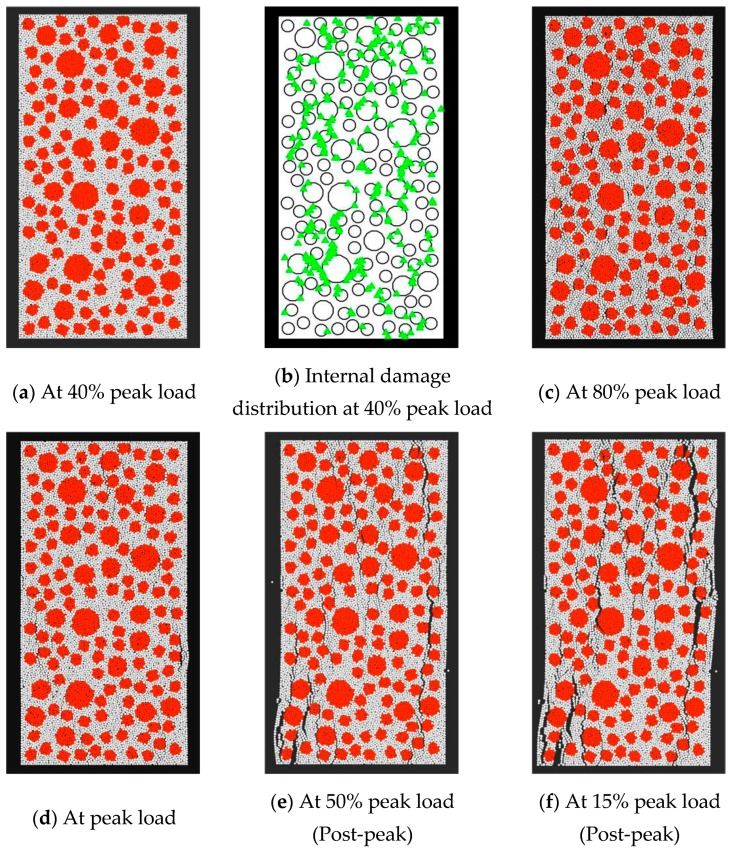
Numerical crack development in ordinary concrete under compression.

Figure 5 displays the numerical crack development in concrete under axial compression. The green triangle in Figure 5b highlights tensile failure. The micro-cracking portrayed is mainly attributable to the deformation incompatibility between the coarse aggregate and the cement. This causes tensile failure at the interfacial transition zones. As the load increases, macroscale cracks progressively propagate into the mortar matrix, but their total presence is still low as the applied load approaches to the bearing capacity. Beyond the bearing capacity, the rate of crack propagation increases notably, and the macroscale cracks begin to join into several wide main cracks. The final cracks develop in the vertical direction, and they mainly occur in the matrix phase and the ITZs between aggregate pieces and mortar. Few of them pass directly through the coarse aggregate pieces.

Note, however, that the parameters used for the aggregate–aggregate contact differed significantly from the actual properties of coarse aggregate in a concrete mix. The reason for this disparity may be the simplicity of the constitutive model in the discrete element method. In the simulations of uniaxial tension and compression reported in the literature [52,53,54], the coarse aggregate’s elastic modulus and compressive strength were set at 85.0 GPa and 500.0 MPa, respectively. That is also notably different from the actual properties.

**Figure 6:**
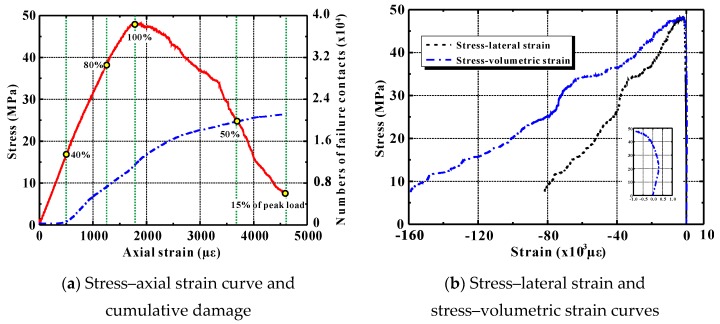
Numerical stress–strain relationships of ordinary concrete under uniaxial compression.

Figure 6a,b depict numerical stress–strain relationships typical of ordinary concrete under axial compression, including the axial strain, the lateral strain, and the volumetric strain. The calculated peak strain (about 1790 *μ*ε) was observed to be a little smaller than the measured one (2039 *μ*ε) [50]. However, on the whole, the discrete element model developed using PFC2D software can simulate the axial deformable responses of the concrete correctly, and it can also correctly capture the lateral deformation of this material.

Figure 6a shows the development of numerous failure contacts under direct compression. The amount of contact failure and the axial deformation generally present an S-shaped relationship. This shape is very similar to that describing cumulative ring deformation in acoustic emission tests [55]. Up to about 40% of the peak load, the discrete element model represents almost no failure contact. This implies that the concrete behaves more or less elastically at that stage. As the applied load increases, contact failure first occurs at the ITZs between the coarse aggregate and the mortar. The number of failed contacts increases sharply between 40% of the peak load and 50% beyond the peak. It then increases more moderately. This nicely reflects the cumulative damage observed in concrete.

### 4.2. Parametric Analysis

A parametric analysis on the mechanical responses of ordinary concrete was conducted in this section. To conveniently compare the numerical results with test results reported in the literature [3], the rectangle’s dimensions were changed to 200 mm × 400 mm, but the mesoscale parameters used in the discrete element model of ordinary concrete remained as the calibrated values.

#### 4.2.1. Influence of the Mortar’s Properties

Previous research [13] showed how the interfacial transition zones change depending on the mortar’s properties. The ratios, however, remain unchanged regardless of the water–cement ratio in ordinary concrete. Thus, the parameter *k* remains at 0.80 during any parametric analysis. Altering the mortar’s macroscale properties is realized through changing its model’s mesoscale parameters, specifically the mortar–mortar contact parameters E^*^ = 21.0 GPa, *σ*_t_ = 5.6 MPa, and *c* = 15 MPa. Those values can be changed proportionately using factors ranging from 0.20 to 1.20 (0.20 is the tolerance). The resulting six concrete rectangles are termed specimens A to F in the discussion below.

**Figure 7:**
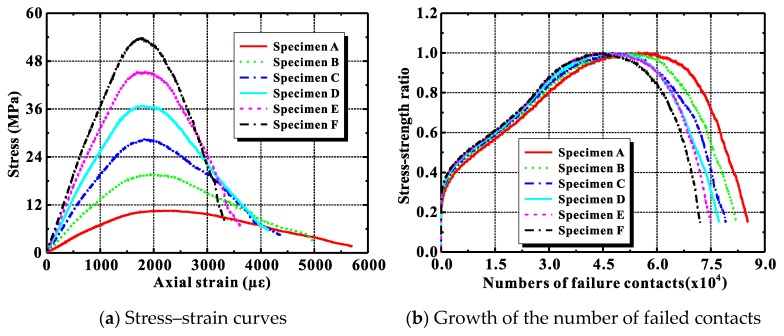
Influence of mortar attribution on compressive behaviors of ordinary concrete.

Figure 7a presents the numerical stress–strain curves in compression for the six concrete rectangles, and Table 5 lists the specimens’ calculated mechanical properties. Changing the mortar’s macroscale properties clearly affected the Poisson’s ratio of ordinary concrete only slightly. It almost always remained at 0.23. The compressive strength, however, increased greatly from 10.56 MPa to 53.85 MPa, while the elastic modulus also increased notably from 7.65 GPa to 39.24 GPa. Note also that the descending proportion of the compressive stress–strain curve became steeper. This implies that the brittleness of the concrete increased. All of these tendencies agree well with the published experimental results [56].

Figure 7b plots the change in the number of failed contacts given by the discrete element simulation. As the mortar’s strength increased, initial contact failure was delayed in the discrete element model, although the total number of failed contacts in the specimen as a whole increased throughout the compressive loading.

#### 4.2.2. Influence of the ITZs

In the process of casting ordinary concrete, if the coarse aggregate’s surface is polluted or the aggregate particles are soaked in certain solutions, the ITZ property of new concrete mix can be influenced greatly. This can affect the details of the ordinary concrete’s mechanical performance in compression.

During the mesoscale simulations, the macroscale attributes of the mortar remained unchanged, and mesoscale E*, *σ*_t_, and *c* remained at 21.0 GPa, 5.6 MPa, and 15.0 MPa. However, the parameter *k* in Table 4 took the values 0.1, 0.2, 0.4, 0.6, 0.8, 1.0, and 1.2. This generated seven concrete samples, denoted as specimens A to G in this section of the discussion.

**Figure 8:**
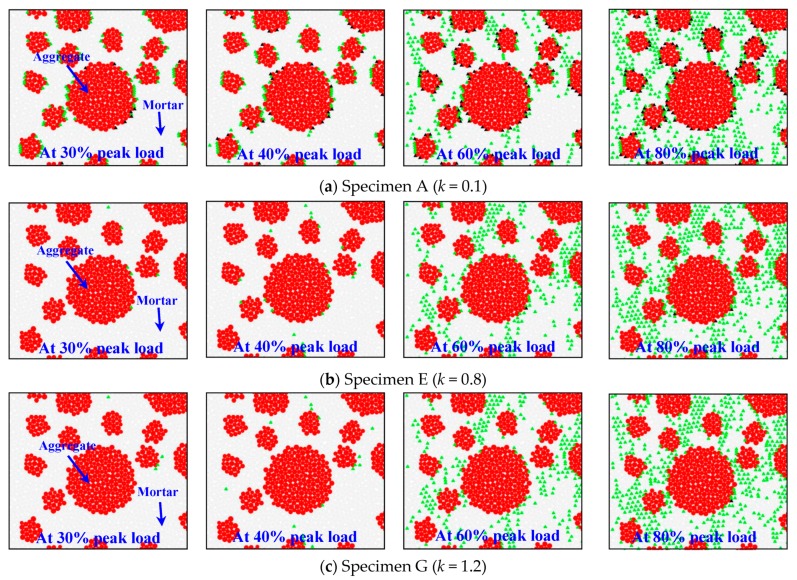
Microscale cracking in ordinary concrete at stress levels of 30% to 80% of the ultimate load by discrete element method (DEM).

As stated in Section 1, the discrete element method can visually express the nonlinear damage and cracking process in the concrete, which are generally hard to observe in traditional mechanical tests. To illustrate this, Figure 8 presents the simulated microscale crack development and distribution in specimens A, E, and G as the applied load reached 30% to 80% of the bearing capacity. In this figure, the green triangle again highlights tensile failure. The black triangle highlights shear failure. Clearly, in specimen A, with an ITZ-to-mortar ratio of 0.1, tensile failures happened frequently in the ITZs between the aggregate and the mortar once the applied load reached 30% of the bearing capacity. At that time shear failures also occurred in some of the ITZs. As the applied load increased further, microscale damage, at first localized at the ITZs, progressively propagated into the mortar matrix. This indicates that the ITZs between the aggregate and the mortar were the weakest zones in the concrete during the initial loading.

Consistent with these observations, in specimen G, tensile failures were fewer and shear failure was never observed up to 40% of the peak load. Upon increasing the applied load to 60% of the bearing capacity, most of the mesoscale damage was in the mortar matrix. At that point, a few contact failures could be observed in the ITZs between the aggregate and the mortar, but their total number was significantly lower than in specimen A. This may imply that the weakest region in specimen G shifted into the mortar phase.

**Figure 9:**
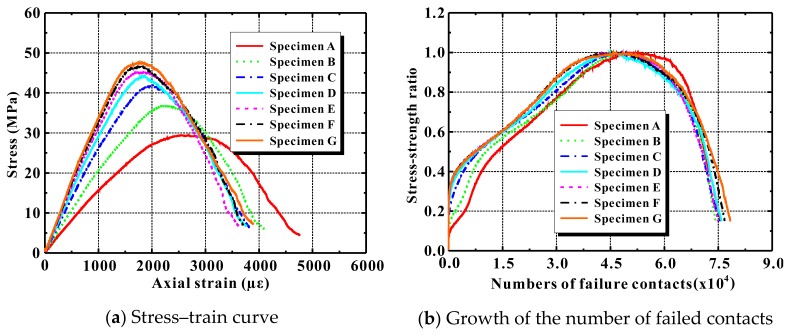
Influence of interfacial transition zones (ITZs) on the compressive behavior of ordinary concrete.

Figure 9a and Table 6 show the numerical compressive stress–strain relationships and the mechanical properties of the seven samples. It is easy to see from Figure 9a that the ITZs played an essential role in determining the compressive mechanical responses of ordinary concrete. When the ITZ attribution was smaller, the stress–strain curve became flatter, and the compressive strength and the elastic modulus were both reduced significantly. However, the peak strain increased sharply. From Table 6, it can be calculated that as the value of *k* decreased from 1.2 to 0.1, the concrete’s strength declined from 47.73 MPa to 29.46 MPa with an amplitude decrease of about 38%. Meanwhile the elastic modulus decreased from 36.64 GPa to 16.17 GPa, a 56% decrease. Obviously, the adverse effect of weak ITZs on the elastic modulus was more significant than that on the compressive strength.

Additionally, taking specimen E as a reference, it is easy to find that an excessive enhancement of the ITZ attribution affected the ordinary concrete’s mechanical properties slightly. As the ITZ-to-mortar ratio *k* increased from 0.8 to 1.2, the growths of the strength and modulus were only 5.4% and 9.4%, respectively. This may have been due to the shift of the weakest region in the concrete. The improvement of the performance of ITZs between the aggregate and the mortar made most of micro-cracks occur at the mortar phase, but the property of this region was little influenced by the interface enhancement method.

Figure 9b further displays the numerical progression of the number of failed contacts during the axial compression of specimens A–G. As can be seen clearly there, when the ITZ-to-mortar ratio *k* was 0.1 or 0.2, microscale cracking began earlier. However, as *k* varied from 0.6 to 1.2, the appearance of the failure of contact remained almost unchanged in specimens D–G.

## 5. Discrete Element Modeling of Recycled Lump Concrete under Compression

### 5.1. Determining the Mesoscale Parameters

The discrete element simulation of recycled lump concrete involves modeling the following eight kinds of contacts:

- New aggregate contacting new aggregate (denoted as new aggregate–new aggregate contact);
- New mortar–new mortar contact;
- New aggregate–new mortar contact;
- Old aggregate–old aggregate contact;
- Old mortar–old mortar contact;
- Old aggregate–old mortar contact;
- Old aggregate–new mortar contact;
- Old mortar–new mortar contact.

The former six contacts describe the new and old concrete. The approach for determining their mesoscale parameters is similar to that illustrated in Table 4. The seventh and eighth types of contacts mainly describe the cohesion between new concrete and the old concrete. The mesoscale parameters employed in describing those contacts are based on the following rules:

(i) The seventh contact mainly reflects the bonding between old aggregate and the new mortar. There should be no notable difference between this type of contact and the aggregate–mortar contact in the new concrete. Therefore, the parameters employed in the seventh contact are the same as those used to represent the third (new aggregate–new mortar) contact.

(ii) The cohesive behavior of the ITZs between the old and new mortar can refer to published test results describing old mortar–new mortar interfaces.

A group led by Xiao published [13] the results of nanoscale indentation tests on recycled aggregate concrete. They showed that the properties of the old mortar–new mortar interface are tightly linked with those of the new mortar. As the microscale modulus of the new mortar varies, the micro-modulus of the old mortar–new mortar ITZs changes in tandem, maintaining the ratio of the ITZ’s modulus to that of the new mortar’s close to 85%. Liu and his colleagues also published [57] the results of similar nanoscale indentation tests. They found that the microscale hardness of the old mortar–new mortar ITZs is the same as the hardness of the new mortar. They attributed this result to the contribution of the recycled coarse aggregate’s characteristics. Recycled aggregate is much rougher than typical natural aggregate, and its surface has many more microscale pores. Both of those features enhance the bonding between the new mortar and old mortar.

In this study’s simulations, the macroscale properties of the new mortar–old mortar contacts were conservatively assumed to be 85% of those of new mortar–new mortar contacts. Table 7 summarizes the detailed parameters of the eight types of contacts used in the discrete element model of the recycled lump concrete.

### 5.2. Crack Development

Traditional measuring techniques have difficulty expressing the initialization and propagation of cracks within concrete, but the DEM can provide a primary representation. In this study, three recycled lump concrete samples under axial compressive loading were simulated. The DCL replacement ratio simulated was 33%, and the characteristic lump size was 75 mm. The compressive strengths of the new and old concrete represented varied, but the strength combinations were as follows:

(1) The strength of the new concrete *f*_pr,_ _new_ was taken as 45.29 MPa, distinctly higher than that of the old concrete *f*_pr,_ _old_—28.43 MPa;

(2) The compressive strengths of both the new and old concrete were taken as 45.29 MPa;

(3) The new concrete’s strength was taken as 28.43 MPa, distinctly lower than that of the old concrete—45.29 MPa.

The details of the mesoscale parameters of the new and old concrete are illustrated in Section 4.2.1.

**Figure 10:**
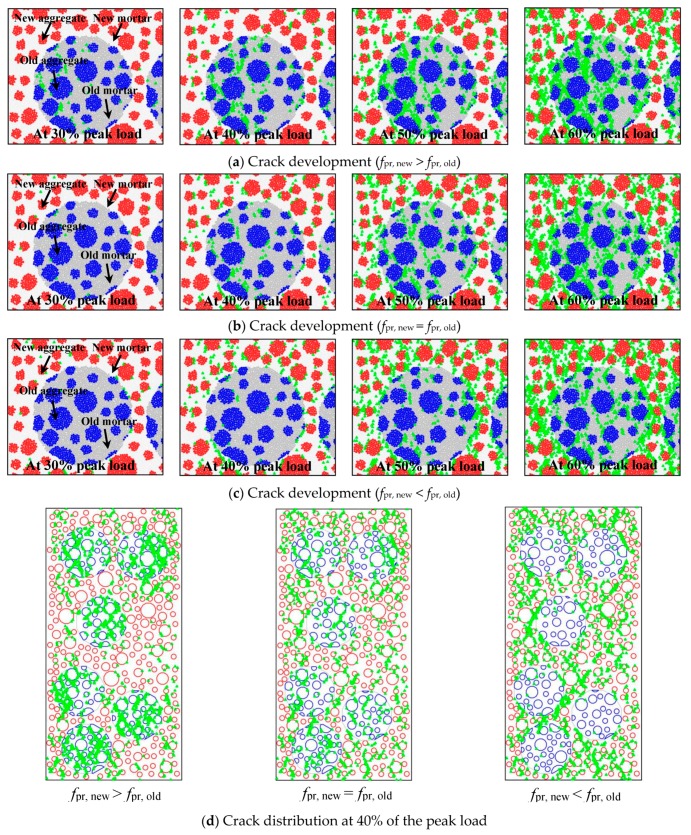
Crack development in recycled lump concrete.

Figure 10a,c present the numerical crack development process around a waste concrete lump when the applied load lies within 30% to 60% of the peak load. The crack distribution for the whole concrete sample at 40% of the bearing capacity given by the DEM is also shown in Figure 10d. The combined strength of the new and old concrete clearly influenced the recycled lump concrete’s behavior in uniaxial compression. When the new concrete was stronger than the old, most of the damage during the initial loading was located at the waste concrete lumps, especially localized at the old aggregate–old mortar interfaces. Failure of the interfaces between new and old concrete also happened, but its amount was relatively small in terms of the overall damage. Thus, the interfaces between the new and old concrete were not an obvious weak point in this situation.

In the case where the new concrete’s strength was equal to that of the old concrete, the crack distribution was relatively uniform over the entire specimen. The amount of damage in the old concrete lumps was much less than in the first case, but the damage in the new concrete was more serious.

When the new concrete was distinctly weaker than the old, the microscale damage was localized in the new concrete, especially at the new aggregate–new mortar interfaces. As compared with the former two strength combinations, the amount of contact failure at the ITZs between the new and old concrete was significantly greater.

### 5.3. Influence of the Distribution of DCLs and Coarse Aggregates 

The influence of the spatial distribution of DCLs and aggregates is discussed in this section using the mesoscale multiphase models together with the discrete element method. However, before studying such an effect, it is vital to determine the throw procedure times. This is because the statistical characteristics of the concrete’s mechanical properties are unstable if too few random throws are simulated. However, if too many random throws are simulated, the computation cost is exorbitant.

**Figure 11:**
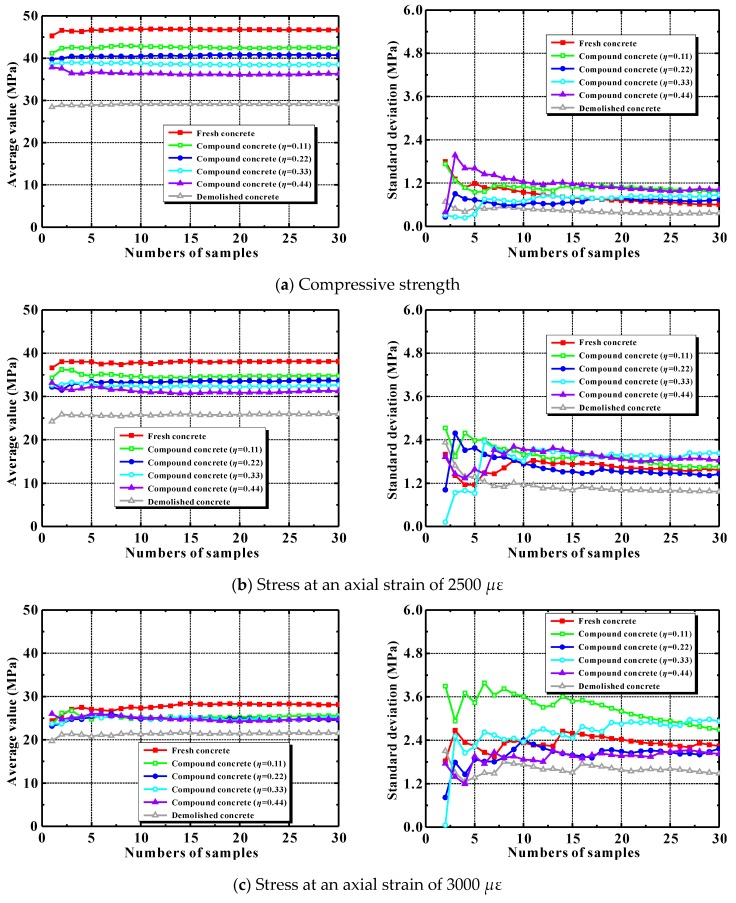
Mean stresses and their standard deviations.

Figure 11 lists the average peak strengths, the stresses at axial strains of 2500 and 3000 *μ*ε, and their standard deviations against the number of random samples. All of the samples in the figure include ordinary concrete, as well as recycled lump concrete with replacement ratios of 11%, 22%, 33%, and 44%. For the recycled lump concrete, the compressive strengths of new and old concrete were taken as 45.29 and 28.43 MPa, respectively (here, it indicates that the new and old concretes adopt the mesoscale parameters of the concretes with the strengths of 45.29 MPa and 28.43 MPa in Table 5). As Figure 11 shows, beyond 20 samples, the concrete average strength and its standard deviation changed only slightly. Thus, the number of random throws was set at 30 in these simulations.

A total of 420 concrete samples were simulated to study the influence of the random distribution of DCLs and coarse aggregates on recycled lump concrete’s performance in compression. The main variables were the relative strength of the new and old concrete, and the lump replacement ratio. Table 8 presents the details of these variables.

**Figure 12:**
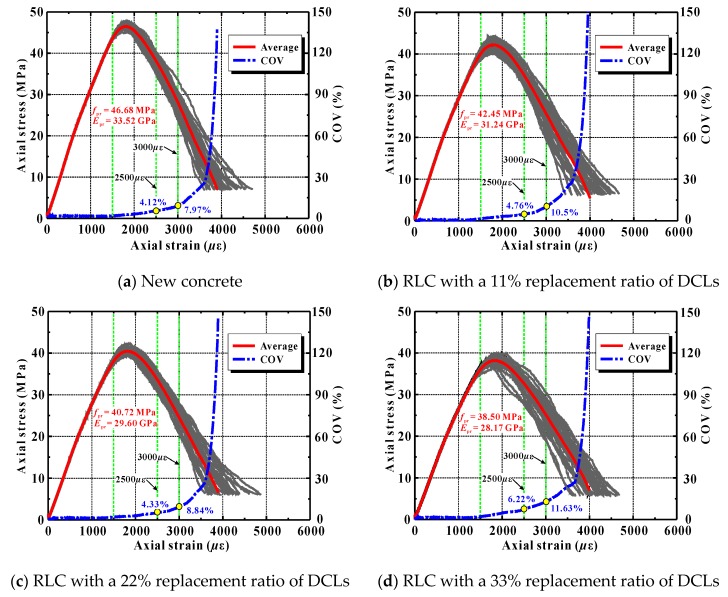

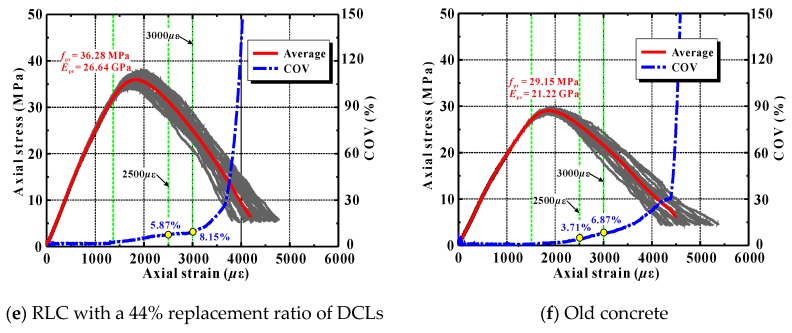
Simulated stress–strain curves of ordinary concrete and recycled lump concrete (RLC).

Figure 12 displays the calculated compressive stress–strain curves of several recycled lump concrete samples. In these samples, the compressive strength of the new concrete was obviously higher than that of old concrete. To better express the influence of the random distribution of DCLs and coarse aggregates on the stress–strain curve, the figure also presents the average stress at full load and its coefficient of variation (COV).

As can be seen from Figure 12, the mean compressive strength and elastic modulus of the recycled lump concrete decreased significantly with increasing lump replacement. This agrees with the observations reported from previous studies [3]. The main reason was due to the incorporation of the weak old concrete. Additionally, it is also easy to see that the variation trend in the stress–strain curve of the recycled lump concrete caused by the random distribution of DCLs and aggregates was similar to that of ordinary concrete induced by the coarse aggregates alone.

(1) Up to 90% of the peak load, the stress–strain curves of the various samples coincided completely, indicating that the random distribution of mesoscale phases affected the compressive performance of the concrete only slightly. It also may be deduced that the elastic modulus was nearly uninfluenced by the spatial locations of the DCLs and coarse aggregate pieces.

(2) As the applied load increased further, an impact of the random distribution of DCLs and coarse aggregates gradually became apparent. The variability of the stress at a specific axial strain also increased.

(3) When the axial load reached 50% to 60% beyond the peak load, the variability of the concrete’s stress–strain curve increased notably and, with continued loading, it continued to increase quickly.

Table 9 shows the statistical results of the peak strength and the stresses at axial strains of 2500 and 3000 *μ*ε for all of the concrete samples listed in Figure 12. Two findings are described below.

(1) The random distribution of coarse aggregates generated variability of about 1.27% in the ordinary concrete’s strength. Du’s group conducted a similar numerical study, and they found that the variability of the concrete’s strength was about 0.91% influences by the spatial location of the coarse aggregates [58]. That agrees well with the present result. However, it should be pointed out that both of those predictions are significantly lower than the tested strength variability from actual experiments (about 5% to 6%) [59,60]. This may because many factors such as initial pores, aggregate shape, and aggregate content influence the compressive strength of concrete, but only the distribution of the coarse aggregates was considered in these simulations.

(2) When the replacement ratios of DCLs were 11%, 22%, 33% and 44%, the corresponding variability in recycled lump concrete’s peak strength was 2.27%, 1.81%, 2.26% and 2.80%, respectively. It can be seen evidently that the strength variability for the recycled lump concrete was generally 0.54–1.53 percentage points greater than that of the ordinary concrete, but this variability had no monotonic trend with an increase in the lump replacement ratio. That higher strength variability of recycled lump concrete may mainly have been due to the coexistence of random distribution of coarse aggregates and random distribution of DCLs. For the same reason, the variability of stress at an axial strain of 2500 or 3000 *μ*ε was also greater for recycled lump concrete than for ordinary concrete.

In the case where the strength of the new concrete was equal to or less than that of the old concrete, the statistical characteristics of concrete stress are presented in Table 9. It is easy to observe the following:

(1) When the two strengths were about equal, the variation in the recycled lump concrete’s compressive strength was relatively small as the replacement ratio of DCLs increased. In that situation, the strength variability of the recycled lump concrete was 0.13–0.27 percentage points stronger than that of ordinary concrete. This was mainly because, even though the attributes of the constituents were equal, the distribution of interfaces between new and old concrete grew with increasing amounts of the DCLs.

(2) When the new concrete was distinctly weaker than the old concrete, the compressive strength of recycled lump concrete increased with the amount of DCLs included. The variabilities of the compressive strength and the stress at an axial strain of 2500 *μ*ε were both slightly higher for recycled lump concrete than for ordinary concrete. However, the variability in the stress at an axial strain of 3000 *μ*ε for recycled lump concrete intercrossed with that for ordinary concrete.

(3) Overall, the strength variability of recycled lump concrete was smallest when the strengths of the new concrete and old concrete were about equal.

The following formulas were proposed in previous studies [3,61] for predicting the compressive strength of recycled lump concrete prisms:

(6) $$f_{\mathrm{pr},\;200,\;\mathrm{RLC}}=(1.0-0.14\times \eta )\times \left[f_{\mathrm{pr},\;200,\;\mathrm{new}}\times (1-\eta )+f_{\mathrm{pr},\;200,\;\mathrm{old}}\times \eta \right],$$

where the subscripts “_RLC_”, “_new_”, and “_old_” denote the recycled lump concrete, the new concrete, and the old concrete, respectively.

Here, Equation (6) was applied to predict the compressive strengths of the 420 recycled lump concrete samples listed in Table 8. The calculated results agree well with the strength values given by the discrete element method. The relative errors were mostly within the range of −7.56% to 8.51%, and the correlation coefficient *R*^2^ was 0.982. The utility of this empirical formulas applied for predicting the compressive strength of recycled lump concrete prisms was re-confirmed in the current discrete element analyses.

### 5.4. The Influence of Lump Shape

A real crushing process produces DCLs with various shapes, but scholarly research mainly focused on roughly spherical lumps. The relevance of its conclusions for other shapes remains to be verified. This study took some steps in that direction through applying the discrete element method.

**Figure 13:**
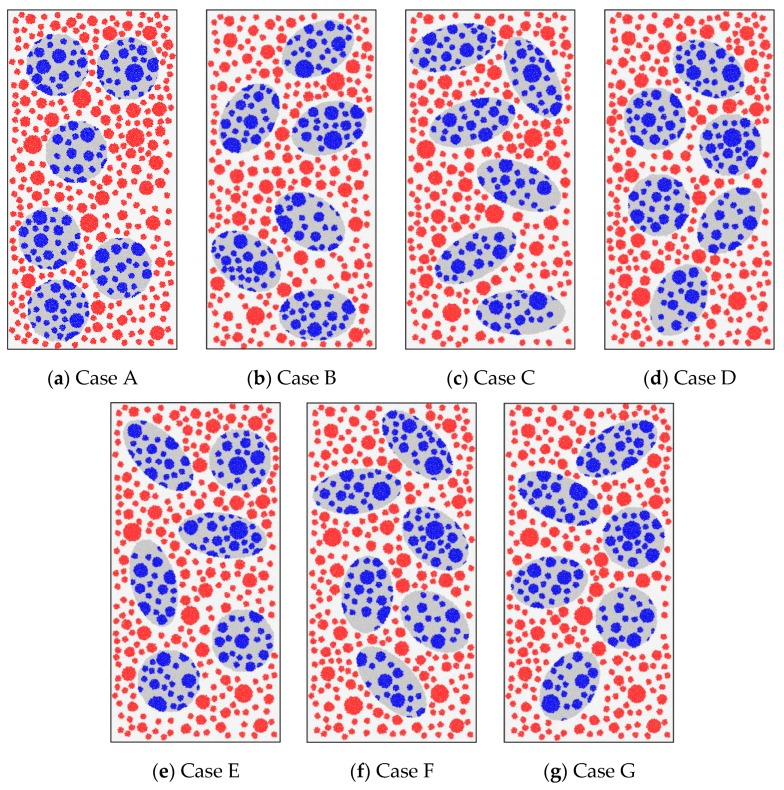
Recycled lump concrete containing DCLs with different shapes.

Figure 13 shows the circular and elliptical DCLs in the simulations. These DCLs had the same area, but they had length ratios of their long axis to short axis of 1.0, 1.5, or 2.0 (see Figure 13a–c). In practice, of course, DCLs with various shapes are used together. The simulations treated four such mixed-use cases (see Figure 13d–g). During these calculations, the compressive strengths of the new and old concrete were taken as 45.29 MPa and 28.43 MPa, respectively (i.e., the new and old concrete adopted the mesoscale parameters of the concretes with the strengths of 45.29 MPa and 28.43 MPa in Table 5). The replacement ratio of DCLs was 33% in all cases. The DCLs and coarse aggregates were thrown 30 times in each case of Figure 13. In this process, the inclination angle of a DCL was not fixed.

**Figure 14:**
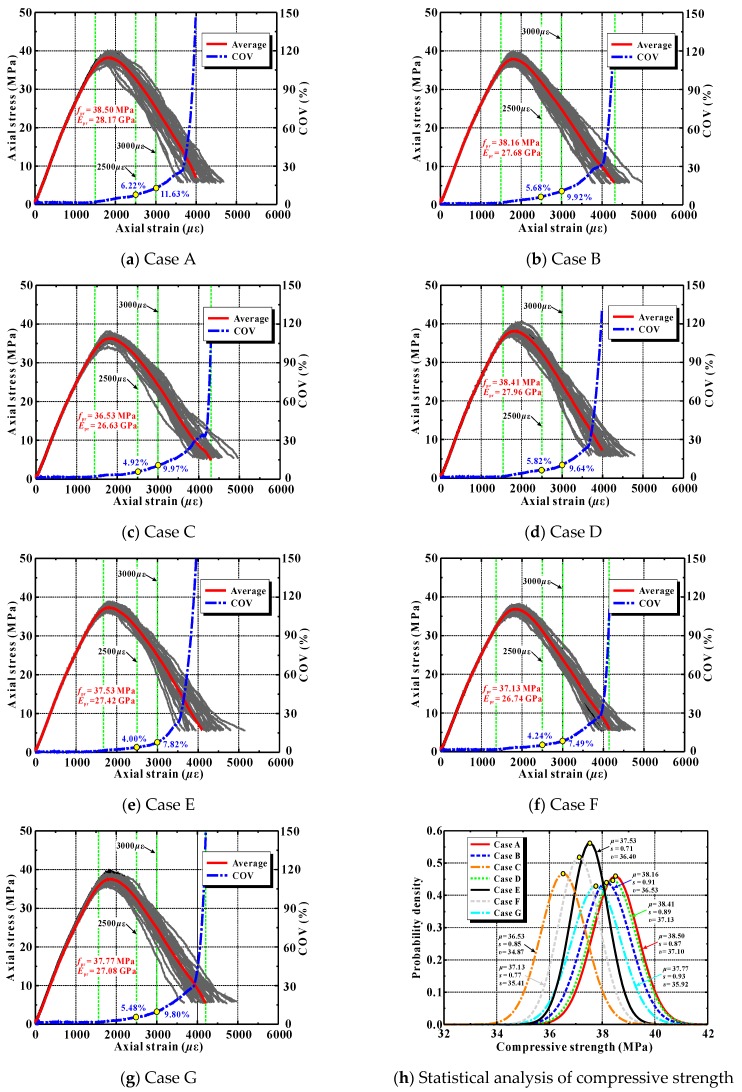
Calculated stress–strain curves for recycled lump concrete containing DCLs with different shapes.

Figure 14 displays the simulated compressive stress–stain curves for the seven cases listed in Figure 13. The mean stress–strain curves and the coefficients of variation for the stresses at axial strains of 2500 and 3000 *μ*ε are also presented. The plots show the following:

(1) The predicted compressive strength and elastic modulus of recycled lump concrete were only slightly influenced by the shape of the DCLs included. The maximum differences for the strength and modulus were only 5.1% and 5.5%, respectively. This is quite different from the results reported in literature [62], which observed that the morphology of the coarse aggregates affects the concrete strength and stiffness notably.

(2) The bonding area between new and old concrete was relatively higher for specimens containing DCLs with a larger length ratio of their long axis to short axis. Comparing the seven cases listed in Figure 13 and Figure 14, the compressive strength and elastic modulus were higher for Cases A and D in which there was less new concrete–old concrete interfacial area. Cases C and F illustrate the situation with a higher number of interfaces, and the mechanical properties were slightly smaller. As illustrated previously, these relationships arose because the old mortar–new mortar interfaces were relatively weak. When casting recycled lump concrete specimens containing small-perimeter DCLs, the interfacial area bonding the old mortar to the new mortar is minimized, which increases the concrete’s strength and elastic modulus.

A Kolmogorov–Smirnov test [58] showed that the compressive strengths for the seven cases conform to the normal distribution. Figure 14h presents the average value *μ*, the variance *s* of the compressive strength, and the strength *v* with their 95% confidence limits. The figure shows that, as the DCL shape changed, the coefficient of variation for the compressive strength fluctuated by 1.89% to 2.46%, but there was no monotonic trend. Therefore, in actual applications of recycled lump concrete, it is feasible to mix in DCLs with a ratio of long axis to short axis smaller than 2.0.

## 6. Recycling Techniques

Currently, there are mainly two methods for recycling waste concrete, forming either recycled aggregate concrete or recycled lump concrete. The former can be considered as a constitutive material replacement. The replacement ratio *ζ* is defined as the ratio of the volume of recycled aggregates to replace the whole volume of natural aggregates. Recycled lump concrete can be treated as a material replacement. The replacement ratio *η* indicates the ratio of the weight of the lumps to the specimen’s total weight (since the densities of the new and old concrete are normally close to each other, the replacement ratios based on the weight and volume fractions can be treated as interchangeable). How do these two recycling techniques differ in terms of the product’s mechanical behavior in compression?

**Figure 15:**
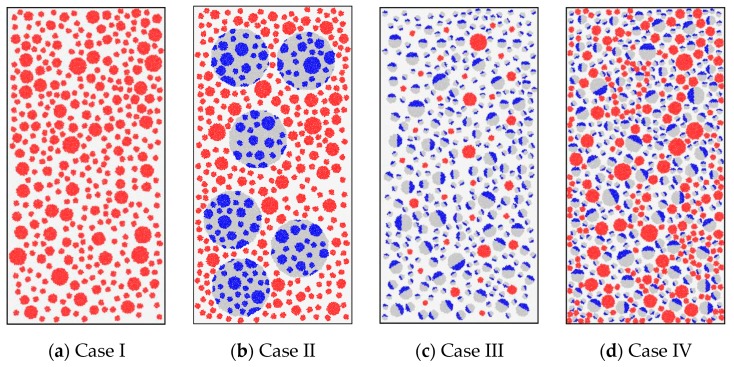
Calculation cases.

Figure 15 displays the four cases simulated in this study. The rectangular samples in Case I were both made entirely with natural aggregates. Their compressive strength was predicted to be about 45.29 MPa. The samples in Case II were recycled lump concrete, and the strengths of the new and old concrete were both set at 45.29 MPa (again, this implies that the new and old concretes adopt the mesoscale parameters of the concrete with the strength of 45.29 MPa in Table 5), the same as in Case I. The replacement ratio of DCLs in Case II was 33%. The Case III samples were made of recycled aggregate concrete. The mesoscale parameters of the new mortar, old mortar, new aggregate, and old aggregate were the same as those in Case II. The amounts of old aggregate and old mortar in this case were also supposed to be the same as those in Case II. This led to a replacement ratio of recycled aggregate of 94.7% and an attached old mortar content in the recycled aggregate of 62.9% according to Equations (7)–(13) (during the calculations, the densities of the coarse natural aggregate and the mortar were assumed to be 2700 and 2100 kg/m^3^, respectively, according to the tested data given in literature [2,50,63]). The samples in Case IV were also recycled aggregate concrete, but they were designed using the equivalent mortar volume (EMV) method [21]. That is, extra coarse aggregates were added to recover the aggregate–mortar ratio of the concrete in Case I. During the discrete element simulation, the coarse aggregates and DCLs in each case were again thrown 30 times, which generated 120 samples.

(7) $$m_{\mathrm{mortar},\;\mathrm{RA}}=m_{\mathrm{mortar},\;\mathrm{DCL}}=\eta m_{\mathrm{concrete}}\frac{0.629\rho_{\mathrm{mortar}}}{0.371\rho_{\mathrm{CA}}+0.629\rho_{\mathrm{mortar}}}.$$

(8) $$m_{\mathrm{CA},\;\mathrm{RA}}=m_{\mathrm{CA},\;\mathrm{DCL}}=\eta m_{\mathrm{concrete}}\frac{0.371\rho_{\mathrm{CA}}}{0.371\rho_{\mathrm{CA}}+0.629\rho_{\mathrm{mortar}}}.$$

(9) $$V_{\mathrm{mortar},\;\mathrm{RA}}=m_{\mathrm{mortar},\;\mathrm{RA}}/\rho_{\mathrm{mortar}}.$$

(10) $$V_{\mathrm{CA},\;\mathrm{RA}}=m_{\mathrm{CA},\;\mathrm{RA}}/\rho_{\mathrm{CA}}.$$

(11) $$\omega_{\mathrm{mortar}}=\frac{V_{\mathrm{mortar},\;\mathrm{RA}}}{V_{\mathrm{CA},\;\mathrm{RA}}+V_{\mathrm{mortar},\;\mathrm{RA}}}.$$

(12) $$\rho_{\mathrm{concrete}}=0.371\rho_{\mathrm{CA}}+0.629\rho_{\mathrm{mortar}}.$$

(13) $$\zeta =\frac{V_{\mathrm{CA},\;\mathrm{RA}}+V_{\mathrm{mortar},\;\mathrm{RA}}}{0.371\frac{m_{\mathrm{concrete}}}{\rho_{\mathrm{concrete}}}}.$$

In Equations (7)–(13), *m*_mortar, RA_ and *m*_CA, RA_ denote the masses of the old mortar and coarse aggregate in the recycled aggregate, respectively. *V*_mortar, RA_ and *V*_CA, RA_ refer to the volumes of the old matrix and old aggregate in the recycled aggregate, respectively. *m*_mortar, DCL_ and *m*_CA, DCL_ denote the masses of old mortar and aggregates in the DCLs, while *m*_concrete_, *V*_concrete_, and *ρ*_concrete_ are the mass, volume, and density of the concrete sample, respectively. The two constants of 0.371 and 0.629 indicate the volume fractions of coarse aggregates and mortar in the concrete mix. They were calculated in accordance with the data presented in Table 1.

**Figure 16:**
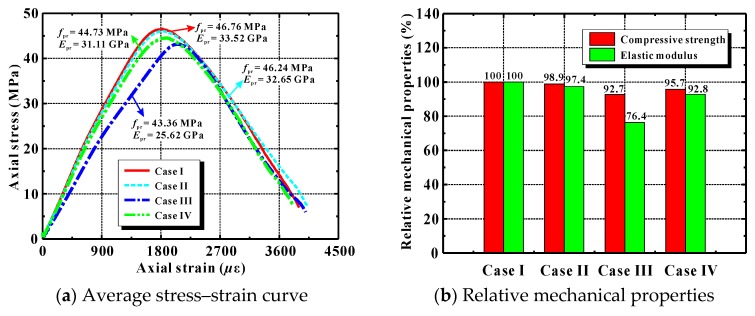
Simulated mechanical performance of various types of concrete in compression.

Figure 16a displays simulated average stress–strain curves for the samples of Cases I–IV. Figure 16b shows the corresponding relative compressive strength and elastic modulus. Three findings are presented below.

(1) The compressive strength and elastic modulus in Case II were 98.9% and 97.4% of those in Case I, respectively. This implies that the compression performance of the recycled lump concrete was slightly inferior to that of ordinary concrete.

(2) The mechanical properties of RAC prepared using a conventional mixing method were normally inferior to those of ordinary concrete. This was shown by the compressive strength and elastic modulus in Case III—only 92.7% and 76.4% of those in Case I. A group led by Jayasuriya published similar simulations [64], and they reported that the strength and modulus of RAC with a replacement ratio of 100% and an old mortar content of 50% were 9% and 22% smaller than those of natural aggregate concrete, respectively. Those results are very close to those predicted here. The main reason for the degradation in RAC’s mechanical properties may be the presence of the old mortar attached on the recycled aggregate. It decreases the aggregate fraction in the concrete, reducing the concrete’s mechanical properties [2].

(3) Using the EMV method increased the compressive strength and elastic modulus in Case IV by 3.1% and 21.4%, respectively, compared to Case III. They were, however, still 4.3% and 7.2% lower than the corresponding values in Case I.

These comparisons show that it is more appropriate to apply the recycled lump concrete or recycled aggregate concrete designed with an EMV method. Using these approaches will promote the application of waste concrete in construction.

## 7. Conclusions

Two-dimensional mesoscale simulations of recycled lump concrete (RLC) samples under compression were performed and analyzed in this study. They were designed to quantify the effects of the strength of the new and old concrete, the replacement ratio of demolished concrete lumps (DCLs), and the DCL distribution and shape on the resulting RLC’s performance under compressive loading. The differences in compression behavior between RLC and recycled aggregate concrete were also predicted.

(1) The study showed that, using the PFC2D discrete element software, two-dimensional, mesoscale, multiphase models of ordinary concrete and RLC can easily be created. Compared with the traditional “take and place” technique, the suggested method does not need any optimization algorithm and, thus, has better generality. A further generalization of the proposed method to three-dimensional, irregular-shape aggregates and DCLs is needed.

(2) The cracking in RLC under axial compression is notably influenced by the strength combination of the new and old concrete. When the two strengths are similar, the distribution of macroscale cracks in the RLC is relatively uniform, and cracks do not localize. However, if there is a significant strength difference between the new and old concrete, micro-cracks usually occur in the concrete’s weaker side, and initiate at the ITZs between aggregate and mortar of the weaker concrete.

(3) The influence tendency of the spatial locations of DCLs and coarse aggregate pieces on the compressive stress–strain curves for RLC is similar to that of the locations of coarse aggregates for ordinary concrete. Regardless of the relative strength of the new and old concrete, the strength variability resulting from random distribution of DCLs and aggregates is more significant for RLC than that for ordinary concrete only induced by random distribution of aggregates. The variability shows no monotonic trend with increasing DCL fraction.

(4) The utility of the empirical formulas currently applied for predicting the compressive strength of RLC prisms was re-confirmed in these discrete element analyses.

(5) The shape of the included DCLs has little influence on the compressive strength and elastic modulus of RLC. In practice, it is feasible to mix DCLs with different shapes as long as the length ratio of their long axis to short axis is smaller than 2.0.

(6) When the strengths of the new and old concrete are similar, both the compressive strength and elastic modulus of RLC are only slightly inferior to those of ordinary concrete. The more appropriate techniques for using waste concrete in construction practice are the RLC or recycled aggregate concrete designed with an equivalent mortar volume method.

## Figures and Tables

**Table 1:** Aggregate size distribution in a mesoscale model of ordinary concrete.

Aggregate Size Range (mm)	Equivalent Aggregate Size (mm)	Aggregate Area Ratio (%)
0–5	Neglected	35.7
5–10	7.5	14.7
10–15	12.5	10.9
15–20	17.5	8
20–25	22.5	3.5

**Table 2:** Parameters adopted in the flat joint model.

Meso-Level Properties	Literature	This Study	Meso-Level Properties	Literature	This Study
Effective modulus E^*^	—	By test	Effective modulus of parallel springs *E*_p_	E*	E*
Stiffness ratio k^*^	2–3.5	3.1	Stiffness ratio of parallel springs *k*_p_	k*	k*
Surface gap *g*_n_	0–0.3	0	Tensile strength *σ*_t_	—	By test
Friction coefficient *μ*_s_	0–0.6	0.6	Cohesion strength *c*	—	By test
Normal damping ratio *β*_n_	0.5	0.5	Friction angle *φ*	0–50°	26.7°
Shear damping ratio *β*_s_	0	0	Radial element *N*	2–4	2

**Table 3:** Mix proportions and mechanical properties of the mortar and concrete.

Specimen	Mix Proportions	Mechanical Property (MPa)			
	Cement:Sand:Gravel:Water	*f* ^test^	*f* ^num^	*E* ^test^	*E* ^num^
Mortar	1:1.23:0:0.21	43.51	42.08	23.55 × 10^3^	23.17 × 10^3^
Concrete	1:1.23:1.85:0.21	49.19	48.30	35.76 × 10^3^	33.41 × 10^3^

Note: The measured mechanical properties of the concrete and the measured strength of the mortar listed in this table were determined by testing six identical samples [50], but the so-called measured elastic modulus of the mortar was calculated through the equation $E_{\mathrm{mortar}}^{\mathrm{test}}$ = 1000(7.7ln($f_{\mathrm{mortar}}^{\mathrm{test}}$) − 5.5).

**Table 4:** Parameters of contacts in discrete element modeling of ordinary concrete.

Contact Parameter	E^*^ (GPa)	*σ*_t_ (MPa)	*c* (MPa)
Mortar–mortar contact	*α*	*β*	*γ*
Aggregate–mortar contact	*kα*	*kβ*	*kγ*
Aggregate–aggregate contact	147	39.2	105

**Table 5:** The ordinary concrete specimens’ mechanical properties.

Specimen	A	B	C	D	E	F
Compressive strength (MPa)	10.56	19.58	28.43	36.85	45.29	53.85
Elastic modulus (GPa)	7.65	14.53	21.06	27.30	33.49	39.24
Poisson’s ratio	0.22	0.23	0.23	0.23	0.23	0.23

**Table 6:** Influence of interfacial transition zones (ITZs) on the mechanical properties of ordinary concrete in compression.

Specimen	A	B	C	D	E	F	G
Compressive strength (MPa)	29.46	36.86	41.90	44.39	45.29	46.63	47.73
Elastic modulus (GPa)	16.17	21.33	27.19	30.80	33.49	35.24	36.64
Poisson’s ratio	0.29	0.25	0.23	0.23	0.23	0.23	0.23

**Table 7:** Parameters of eight types of contacts in recycled lump concrete.

Region	Contact Type	E^*^ (GPa)	*σ*_t_ (MPa)	*c* (MPa)
New concrete	New aggregate–new aggregate contact	147	39.2	105
	New mortar–new mortar contact	*α*_new_	*β*_new_	*γ*_new_
	New aggregate–new mortar contact	0.8*α*_new_	0.8*β*_new_	0.8*γ*_new_
Old concrete	Old aggregate–old aggregate contact	147	39.2	105
	Old mortar–old mortar contact	*α*_old_	*β*_old_	*γ*_old_
	Old aggregate–old mortar contact	0.8*α*_old_	0.8*β*_old_	0.8*γ*_old_
ITZs between newand old concrete	Old aggregate–new mortar contact	0.8*α*_new_	0.8*β*_new_	0.8*γ*_new_
	Old mortar–new mortar contact	0.85*α*_new_	0.85*β*_new_	0.85*γ*_new_

**Table 8:** Parameter values simulated. DCL—demolished concrete lump.

Parameter	Replacement Ratio of DCLs	Compressive Strengths of New Concrete and Old Concrete
Value	0, 11%, 22%, 33%, 44%, and 100%	*f*_pr, new_ = 45.29 MPa, *f*_pr, old_ = 28.43 MPa
		*f*_pr, new_ = *f*_pr, old_ = 45.29 MPa
		*f*_pr, new_ = 28.43 MPa, *f*_pr, old_ = 45.29 MPa

**Table 9:** Statistical characteristics of concrete stress. COV—coefficient of variation.

Strength Combination	*Η* (%)	Compressive Strength		Stress at a Axial Strain of 2500 *μ*ε		Stress at a Axial Strain of 3000 *μ*ε	
		Avg ± SD (MPa)	COV (%)	Avg ± SD (MPa)	COV (%)	Avg ± SD (MPa)	COV (%)
*f*_pr, new_ > *f*_pr, old_	New	46.68 ± 0.60	1.29	38.11 ± 1.57	4.12	28.11 ± 2.24	7.97
	11	42.45 ± 0.96	2.27	34.84 ± 1.66	4.76	25.62 ± 2.69	10.50
	22	40.72 ± 0.74	1.81	33.61 ± 1.46	4.33	24.51 ± 2.17	8.84
	33	38.50 ± 0.87	2.26	32.66 ± 2.03	6.22	25.15 ± 2.93	11.63
	44	36.28 ± 1.02	2.80	31.25 ± 1.83	5.87	24.82 ± 2.02	8.15
	Old	29.15 ± 0.37	1.25	25.96 ± 0.96	3.71	21.54 ± 1.48	6.87
*f*_pr, new_ = *f*_pr, old_	New (Old)	46.68 ± 0.60	1.29	38.11 ± 1.57	4.12	28.11 ± 2.24	7.97
	11	46.35 ± 0.66	1.42	37.16 ± 1.89	5.07	27.90 ± 2.26	8.10
	22	46.30 ± 0.72	1.56	37.03 ± 1.62	4.37	27.65 ± 2.30	8.32
	33	45.94 ± 0.66	1.44	37.96 ± 1.74	4.59	27.52 ± 2.51	9.13
	44	45.08 ± 0.68	1.51	37.13 ± 1.93	5.19	27.33 ± 2.24	8.19
*f*_pr, new_ < *f*_pr, old_	New	29.15 ± 0.37	1.25	25.96 ± 0.96	3.71	21.54 ± 1.48	6.87
	11	29.83 ± 0.41	1.38	26.34 ± 1.16	4.40	22.52 ± 1.60	7.10
	22	30.77 ± 0.54	1.74	27.37 ± 1.15	4.20	23.43 ± 1.72	7.34
	33	32.00 ± 0.65	2.02	29.16 ± 1.23	4.23	24.00 ± 1.57	6.54
	44	33.17 ± 0.64	1.93	29.63 ± 1.35	4.56	24.94 ± 1.87	7.50
	Old	46.68 ± 0.60	1.29	38.11 ± 1.57	4.12	28.11 ± 2.24	7.97
